# Cannabidiol Attenuates Methamphetamine-Induced Autophagy in Primary Rat Neurons via the 5-HT1A/AC/cAMP/PKA/CREB Signaling Pathway

**DOI:** 10.3390/ijms27135677

**Published:** 2026-06-24

**Authors:** Xiong Li, Jiameng Ding, Xiao Ma, Dongxian Zhang

**Affiliations:** School of Forensic Medicine, National Health Commission Key Laboratory of Drug Addiction Medicine, Kunming Medical University, Kunming 650500, China; lixiong962464@163.com (X.L.); 15121906630@163.com (J.D.); maxiao202507@163.com (X.M.)

**Keywords:** methamphetamine, cannabidiol, neurotoxicity, autophagy, 5-HT1A receptor

## Abstract

Methamphetamine (METH) induces neurotoxicity via excessive and incomplete autophagy, although the underlying mechanisms remain unclear. This study investigated cannabidiol (CBD)’s protective effect and the role of the 5-Hydroxytryptamine 1A receptor (5-HT1A)/adenylyl cyclase (AC)/cyclic adenosine monophosphate (cAMP)/protein kinase A (PKA)/cAMP response element-binding protein (CREB) pathway in primary hippocampal neurons. METH (2 mM, 24 h) reduced neuronal viability, downregulated 5-HT1A, activated the AC/cAMP/PKA/CREB pathway, and simultaneously upregulated autophagy-related proteins (Beclin-1, Microtubule-associated protein 1 light chain 3 [LC3], and Sequestosome 1 [p62]) and overall autophagic flux, indicating impaired lysosomal degradation during autophagy. CBD (1–10 μM) reversed METH-induced autophagy, restored viability, and normalized pathway protein expression. 5-HT1A agonist eptapirone synergized with CBD to inhibit autophagy, while the antagonist WAY-100635 abolished CBD’s effects. These findings demonstrate that CBD, acting as an allosteric modulator of 5-HT1A, alleviates METH-induced neuroautophagy by restoring 5-HT1A activity and suppressing excessive AC/cAMP/PKA/CREB activation, highlighting its potential as a therapeutic agent for METH-related neurotoxicity.

## 1. Introduction

Methamphetamine (METH) is a highly addictive synthetic stimulant with potent central euphoric effects, and one of the most widely abused drugs globally. It is frequently misused as a psychostimulant [1], constituting a major global public health issue [2]. Long-term use of METH can lead to drug dependence and severe neurotoxic effects [3,4,5], inducing abnormal compensatory adaptations in neurons and resulting in numerous irreversible pathological and chemical damage to the nervous system. The toxic damage caused by METH can affect multiple brain regions, including the hippocampus, striatum, prefrontal cortex, and amygdala [6,7]. Studies have found that autophagy in organisms is involved in the development of METH-induced toxic effects, and autophagy activation is a key process in METH-induced neurotoxicity [8]. However, the exact mechanisms remain unclear.

Autophagy is a core molecular pathway for the preservation of cellular and organismal homeostasis. It refers to the process by which cells selectively or non-selectively remove damaged proteins and organelles [9]. Autophagy is central to cellular function and survival, while autophagy and mitophagy pathways are crucial for central nervous system (CNS) development, health, and function [10]. Studies have revealed that the process of autophagy is considerably heightened during METH-induced toxicity, which exacerbates both METH-induced neurotoxicity and its related dependency effects [11]. Mature primary hippocampal neurons are terminally differentiated cells with extremely low basal autophagic activity under physiological conditions, which leads to barely detectable expression of LC3 (microtubule-associated protein light chain 3, a marker of autophagy). METH increases the expression levels of core autophagy-related proteins, especially Beclin-1 (autophagy related gene 6 [ATG6] family) and LC3-II (the major mammalian homolog of ATG8 family), which are classic markers for autophagosome nucleation and elongation respectively [12]. Research has indicated that the activation of 5-hydroxytryptamine (5-HT) receptors suppresses autophagy, leading to a decrease in the expression of Beclin-1 and the LC3-II/LC3-I ratio; conversely, antagonizing 5-HT receptors enhances autophagy [13]. However, it remains unclear whether 5-HT signaling is involved in METH-induced autophagy.

To explore the underlying mechanism, we focused on 5-HT-related signaling pathways, as 5-HT has been linked to autophagy regulation. The G protein/adenylate cyclase (AC)/cyclic adenosine monophosphate (cAMP)/protein kinase A (PKA) signaling pathway is the classical regulatory pathway for cAMP response element-binding protein (CREB) [14]. 5-HT is an important neurotransmitter in the brain; among its subtypes, the 5-HT1A receptor belongs to the superfamily of G protein-coupled receptors (GPCRs) and is negatively coupled to cAMP production [15,16]. The AC/cAMP/PKA/CREB signaling pathway is a downstream molecular pathway of 5-HT1A and is involved in various physiological and pathological processes [17]. It has been demonstrated that PKA not only regulates autophagy but also receives signals from autophagy [18]. Relevant research indicates that the PKA/CREB cascade may also contribute to the formation of autophagic flux [19]. However, the involvement of the 5-HT1A/AC/cAMP/PKA/CREB signaling pathway in METH-induced autophagy remains unclear.

Currently, there are no specific therapeutic medications for METH-induced neurotoxicity. Cannabidiol (CBD), a component extracted from the cannabis plant, does not produce psychoactive effects. CBD exerts multiple biological effects, including immunosuppression, anti-inflammatory, and anti-fibrosis [20,21,22,23]. Research findings indicate that CBD modulates brain dopamine levels in response to substance abuse, thereby reducing drug use and drug-seeking behaviors [24]. Several studies have also shown that CBD intervention can effectively inhibit the behavioral effects in METH-dependent rats [25] and has the potential to diminish the rewarding effects of METH [26]. However, it remains unclear whether CBD exerts protective effects against METH-induced neuronal autophagy.

In the present study, we evaluated the protective effect of CBD against METH-induced autophagy in primary neurons and examined the expression of key factors in the 5-HT1A/AC/cAMP/PKA/CREB signaling pathway. While the 5-HT1A-associated serotonergic mechanism and CBD’s neuroprotective effects have been widely reported, this study explores the linkage between the 5-HT1A/AC/cAMP/PKA/CREB pathway and METH-triggered neuronal autophagy. These findings may provide new strategies for the treatment of neurotoxicity induced by METH abuse.

## 2. Results

### 2.1. METH Induces Autophagy in Primary Neurons

**Figure 1:**
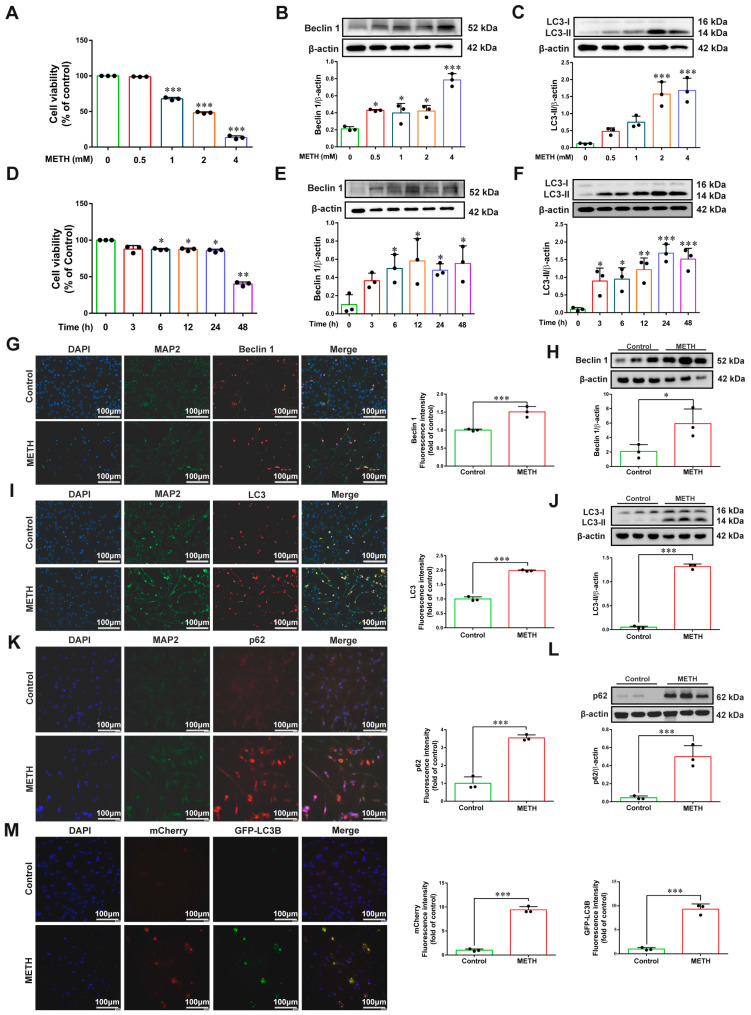
Methamphetamine induces autophagy in primary neurons. (**A**) Cell viability of primary neurons incubated with METH at concentrations of 0, 0.5, 1, 2, 4 mM for 24 h. (**B**,**C**) Western blot analysis and quantitative statistics of Beclin-1 and LC3 protein expression in neurons treated with gradient concentrations of METH for 24 h. (**D**) Cell viability of neurons treated with 2 mM METH for 0, 3, 6, 12, 24, 48 h. (**E**,**F**) Western blot analysis and quantitative statistics of Beclin-1 and LC3 protein expression in neurons treated with 2 mM METH for different durations. (**G**–**L**) Immunofluorescence staining (**G**,**I**,**K**) and Western blot (**H**,**J**,**L**) detection of Beclin-1, LC3 and p62 expression in neurons after 2 mM METH treatment for 24 h; neuronal marker MAP2 and nuclear marker DAPI were used for co-localization. (**M**) Immunofluorescence detection of autophagic flux using Ad-mCherry-GFP-LC3B adenovirus transfection in METH-treated neurons. Scale bar = 100 μm. Results are presented as means ± SD of three experiments. * *p* < 0.05, ** *p* < 0.01, *** *p* < 0.001 vs. Control group; one-way ANOVA followed by Tukey HSD post hoc test or independent samples *t*-test.

After exposure of primary neurons to different concentrations of METH (0, 0.5, 1, 2, and 4 mM) for 24 h, results showed that METH induced a decrease in neuronal viability (Figure 1A) and upregulated the expression of Beclin 1 and LC3 in a concentration-dependent manner (Figure 1B,C). Additionally, treatment with 2 mM METH induced a reduction in neuronal viability (Figure 1D), and upregulated the expression of Beclin 1 and LC3 in a time-dependent manner across the tested time points (0, 3, 6, 12, 24, and 48 h) (Figure 1E,F). Based on these findings, 2 mM METH treatment for 24 h was selected as the autophagy induction condition for subsequent experiments. Further detection of autophagy-related proteins revealed that both immunofluorescence and Western blot results confirmed that 2 mM METH significantly induced neuronal autophagy (with increased expression of Beclin 1, LC3, and p62) (Figure 1G–L). Meanwhile, autophagic flux expression was upregulated following transfection with Ad-mCherry-GFP-LC3B (Figure 1M).

### 2.2. Methamphetamine Can Induce Changes in the Expression of Components Within the 5-HT1A/AC/cAMP/PKA/CREB Signaling Pathway

**Figure 2:**
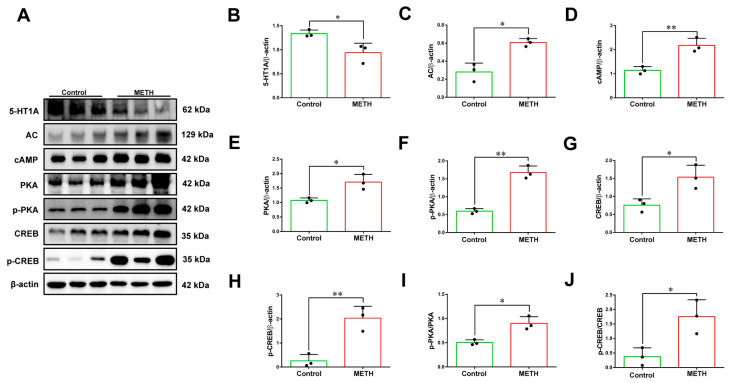
METH induces alterations in the expression levels of pathway-related proteins in primary neurons. (**A**–**J**) Western blot analysis and quantitative quantification of 5-HT1A, AC, cAMP, PKA, p-PKA, CREB and p-CREB protein expression in primary neurons after 2 mM METH treatment for 24 h. β-actin was used as internal reference. Results are presented as means ± SD of three experiments. * *p* < 0.05, ** *p* < 0.01 vs. Control group; independent samples *t*-test.

Subsequently, we analyzed the expression of proteins involved in the 5-HT1A/AC/cAMP/PKA/CREB signaling pathway using Western blot. The results showed that compared with the control group, the METH-treated group exhibited decreased 5-HT1A protein expression, while the expression levels of AC, cAMP, PKA, phosphorylated protein kinase A (p-PKA), CREB, and phosphorylated cyclic AMP response element-binding protein (p-CREB) proteins were upregulated (Figure 2A–H). Additionally, the ratios of p-PKA/PKA and p-CREB/CREB were significantly increased (Figure 2I,J). These findings indicate that METH treatment activates the AC/cAMP/PKA/CREB signaling pathway by downregulating 5-HT1A protein expression.

### 2.3. Intervention on 5-HT1A Can Affect Methamphetamine-Induced Autophagy and Alter the Expression of Pathway Molecules

**Figure 3:**
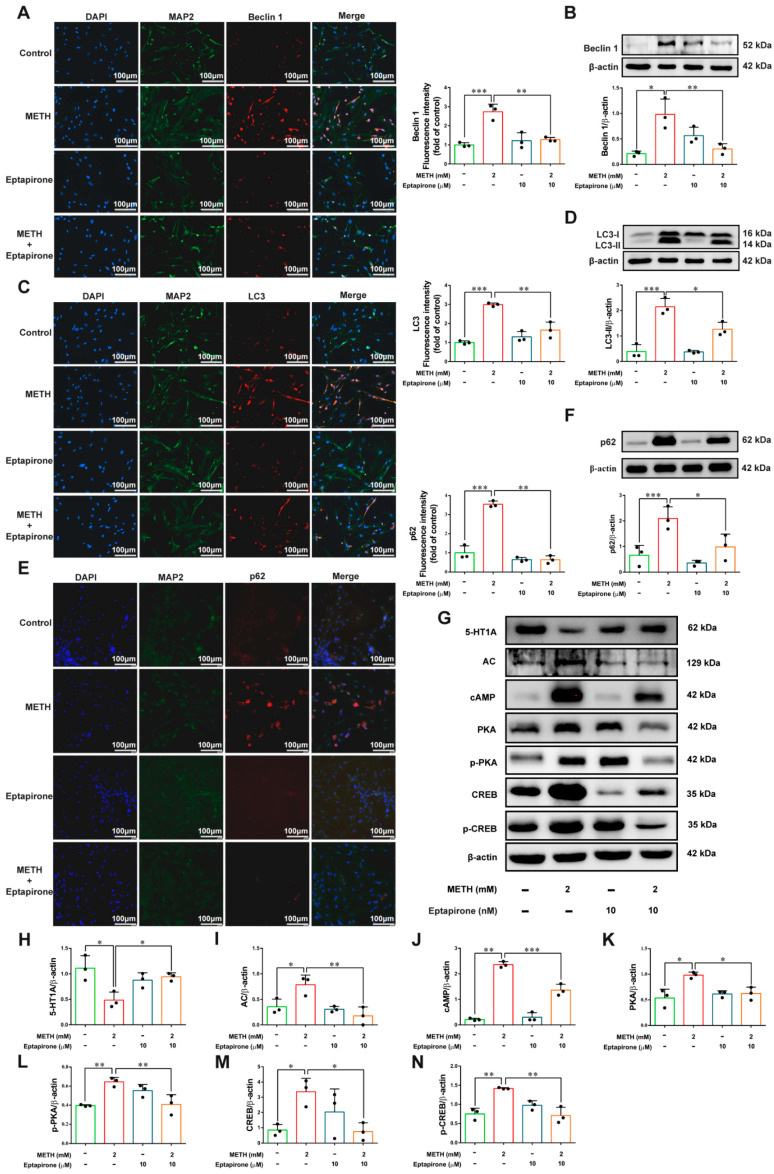
Activation of 5-HT1A receptors attenuates METH-induced autophagy and alters the expression of pathway molecules in primary neurons. (**A**–**F**) Immunofluorescence staining and Western blot analysis of Beclin-1, LC3 and p62 expression. (**G**–**N**) Western blot detection and quantitative analysis of 5-HT1A, AC, cAMP, PKA, p-PKA, CREB and p-CREB protein levels. Scale bar = 100 μm. Results are presented as means ± SD of three experiments. * *p* < 0.05, ** *p* < 0.01, *** *p* < 0.001; one-way ANOVA followed by Tukey HSD post hoc test.

**Figure 4:**
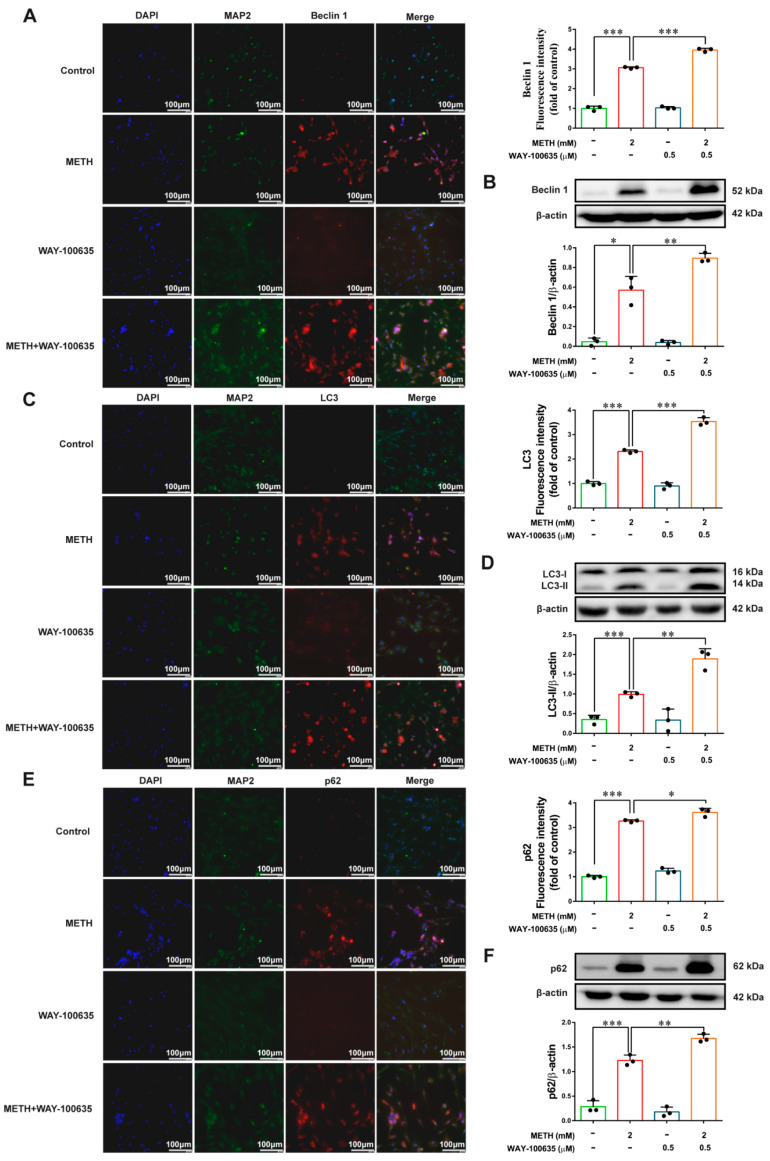
Antagonism of 5-HT1A receptors exacerbates METH-induced autophagy in primary neurons. (**A**–**F**) Immunofluorescence staining and Western blot analysis of Beclin-1, LC3 and p62 expression. Scale bar = 100 μm. Results are presented as means ± SD of three experiments. * *p* < 0.05, ** *p* < 0.01, *** *p* < 0.001; one-way ANOVA followed by Tukey HSD post hoc test.

Eptapirone, a selective 5-HT1A receptor agonist [27], was administered, and subsequent detection of autophagy-related proteins revealed the following: compared with the control group, METH treatment upregulated the expression of Beclin 1, LC3, and Sequestosome 1 (p62), whereas Eptapirone reversed these upregulatory effects (Figure 3A–F). Immunofluorescence results were consistent with those of Western blot. It also reversed the expression of pathway proteins, specifically upregulating the expression of 5-HT1A protein while downregulating the expression of AC, cAMP, PKA, p-PKA, CREB, and p-CREB proteins (Figure 3G–N). Furthermore, when the 5-HT1A receptor antagonist WAY-100635 was administered [28], both immunofluorescence and Western blot analyses demonstrated that WAY-100635 further exacerbated METH-induced autophagy (Figure 4A–F). These research findings indicate that the 5-HT1A signaling pathway plays a critical role in methamphetamine-induced autophagy in primary neurons.

### 2.4. CBD Alleviates METH-Induced Autophagy in Primary Neurons

**Figure 5:**
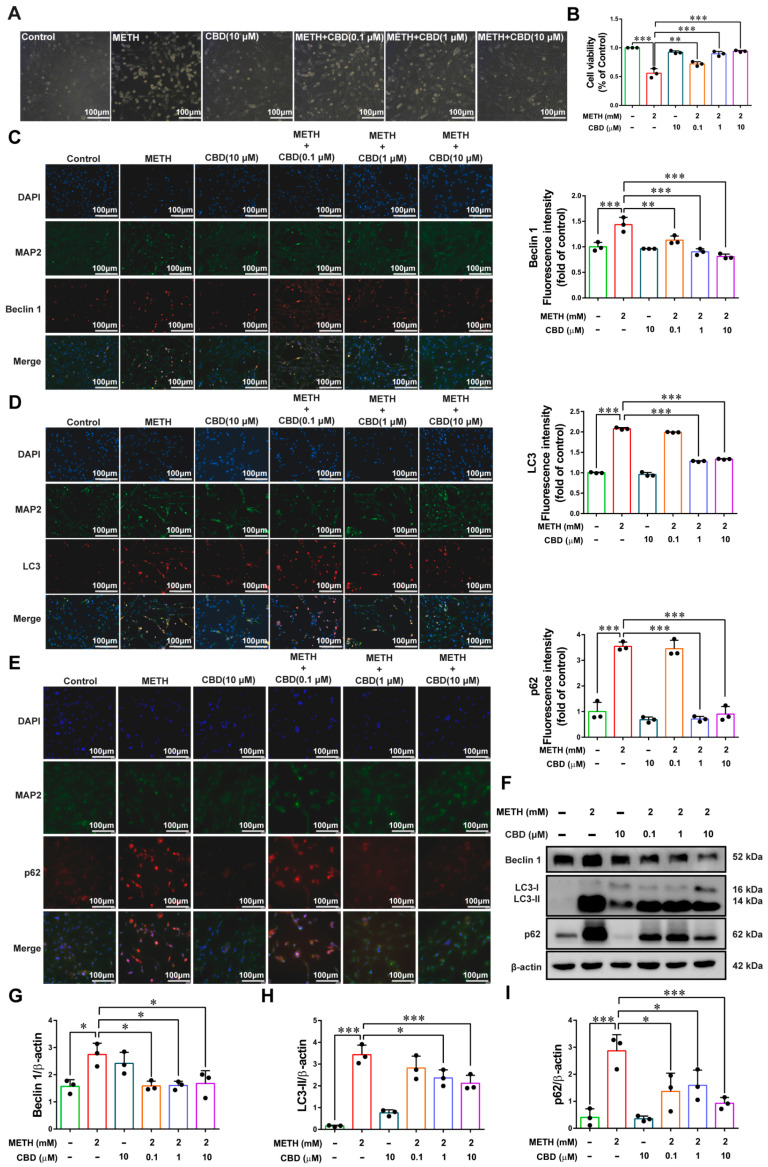
CBD attenuates METH-induced autophagy in primary neurons. (**A**,**B**) Cell viability detected by CCK-8 assay. (**C**–**E**) Immunofluorescence staining of Beclin-1, LC3 and p62. (**F**–**I**) Western blot analysis and quantitative statistics of autophagy-related proteins. Scale bar = 100 μm. Results are presented as means ± SD of three experiments. * *p* < 0.05, ** *p* < 0.01, *** *p* < 0.001; one-way ANOVA followed by Tukey HSD post hoc test.

**Figure 6:**
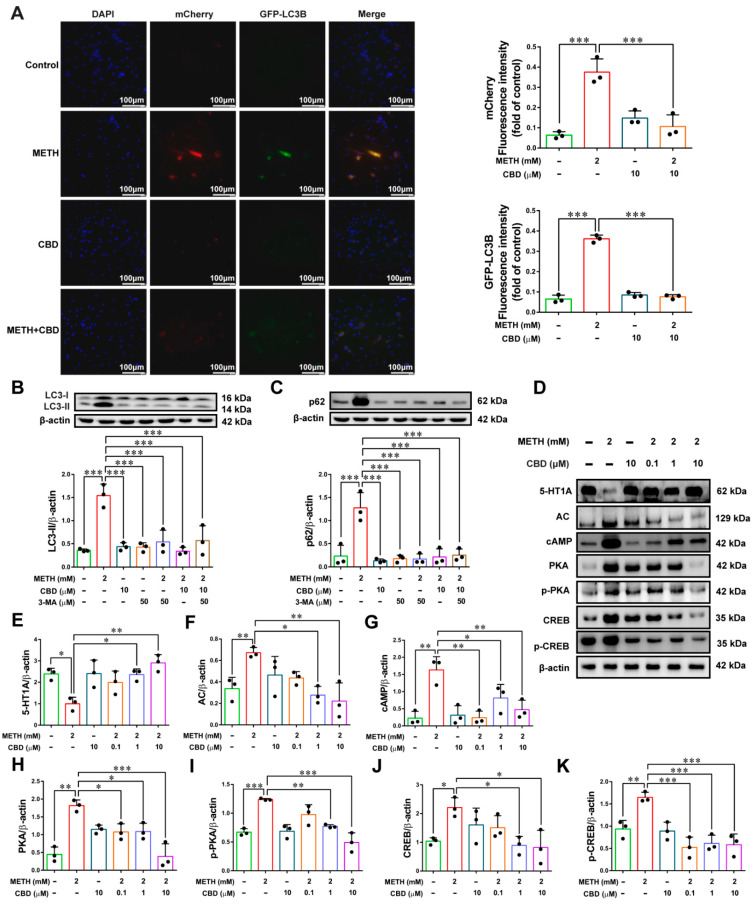
CBD attenuates METH-induced autophagy and alters the expression of pathway molecules in primary neurons. (**A**) Immunofluorescence detection of autophagic flux via Ad-mCherry-GFP-LC3B transfection in METH + 10 μM CBD-treated neurons. (**B**,**C**) Western blot analysis of LC3 and p62 expression after intervention with the autophagy inhibitor 3-MA or CBD. (**D**–**K**) Western blot detection and quantification of 5-HT1A/AC/cAMP/PKA/CREB pathway proteins after CBD treatment. Scale bar = 100 μm. Results are presented as means ± SD of three experiments. * *p* < 0.05, ** *p* < 0.01, *** *p* < 0.001; one-way ANOVA followed by Tukey HSD post hoc test.

We performed post-intervention treatment with CBD and detected neuronal viability using the cell counting kit-8 (CCK-8) assay. The results showed that CBD had no toxicity and could restore neuronal viability (Figure 5A,B). Subsequently, we examined the expression of autophagy-related proteins. The results indicated that treatment with 0.1 μM, 1 μM, and 10 μM CBD all significantly downregulated the expression of Beclin 1 (Figure 5C,F,G). Only 1 μM and 10 μM CBD could significantly reduce the expression of LC3, while 0.1 μM CBD had no significant effect, and the Western blot results were completely consistent with the immunofluorescence results (Figure 5D,F,H). However, all concentrations of CBD were able to decrease the expression of p62 (Figure 5E,F,I). After transfection with Ad-mCherry-GFP-LC3B, we evaluated the effect of CBD on autophagic flux, and the results showed that CBD could significantly reduce the expression of mCherry-GFP-LC3B (Figure 6A). In addition, we applied the autophagy inhibitor 3-Methyladenine (3-MA) [29] and detected the expression of LC3 and p62. The results demonstrated that 3-MA could significantly inhibit the METH-induced increase in autophagy levels, and the effects of CBD were consistent with those of 3-MA (Figure 6B,C).

The above research results preliminarily indicate that CBD can alleviate methamphetamine-induced neuronal autophagy, suggesting that CBD has certain therapeutic value for METH-induced autophagy.

### 2.5. CBD Impairs the Action of METH on 5-HT1A/AC/cAMP/PKA/CREB Pathway Proteins

Subsequently, we detected the protein expression of the 5-HT1A/AC/cAMP/PKA/CREB signaling pathway using Western blot. Consistent with previous findings, compared with the control group, METH decreased 5-HT1A protein expression and upregulated the expression of AC, cAMP, PKA, p-PKA, CREB, and p-CREB proteins (Figure 6D–K). However, following CBD intervention, CBD reversed METH-induced changes in the pathway proteins: it upregulated 5-HT1A expression and downregulated the expression of AC, cAMP, PKA, p-PKA, CREB, and p-CREB proteins. Among these, both 1 μM and 10 μM CBD exerted significant effects, whereas 0.1 μM CBD only downregulated cAMP, PKA and p-CREB proteins (Figure 6D–K). These results indicate that CBD reverses METH-induced alterations in proteins related to the 5-HT1A/AC/cAMP/PKA/CREB signaling pathway, suggesting that CBD may exert its anti-METH effects through this signaling pathway.

### 2.6. Intervention of 5-HT1A Can Alter the Effect of CBD on METH Toxicity

**Figure 7:**
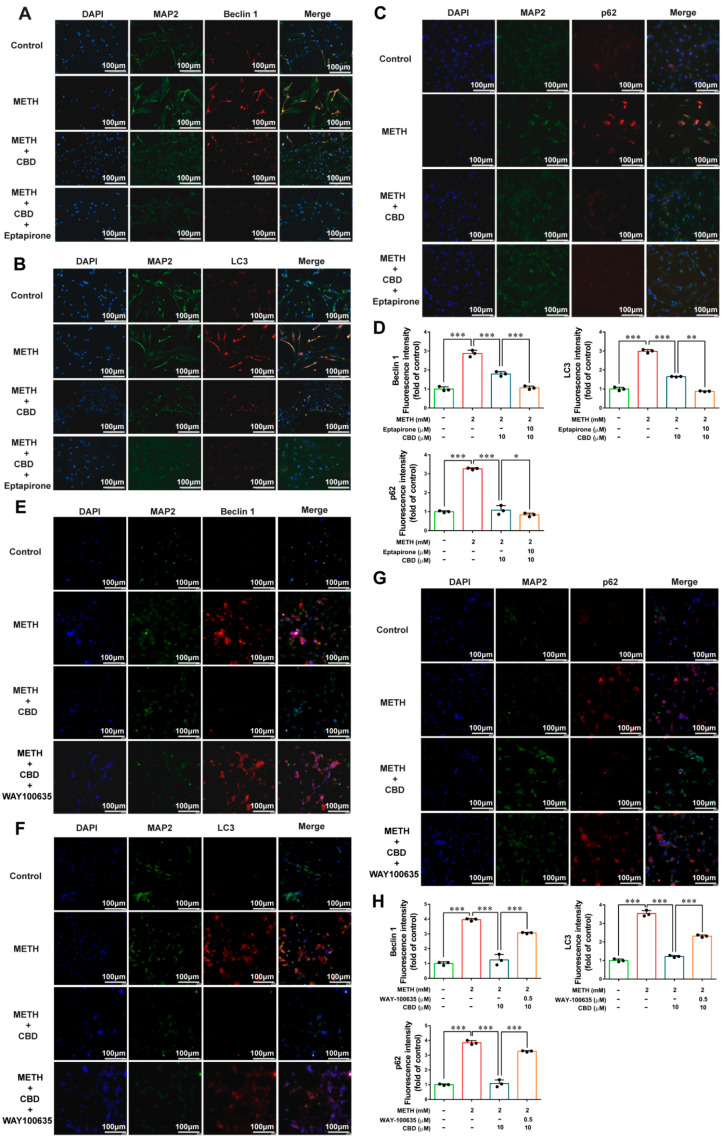
Intervention of 5-HT1A can alter the effect of CBD on METH toxicity. (**A**–**D**) Neurons were pretreated with 10 μM eptapirone, followed by METH and CBD treatment; immunofluorescence staining of Beclin-1, LC3 and p62. (**E**–**H**) Neurons were pretreated with 0.5 μM WAY-100635, followed by METH and CBD treatment; immunofluorescence staining of autophagy-related proteins. Scale bar = 100 μm. Results are presented as means ± SD of three experiments. * *p* < 0.05, ** *p* < 0.01, *** *p* < 0.001; one-way ANOVA followed by Tukey HSD post hoc test.

Prior to METH administration, cells were first treated with Eptapirone or WAY-100635, followed by post-intervention with CBD, and the expression of autophagy-related proteins was detected. Compared with the METH group, the METH + CBD group showed significantly decreased expression levels of Beclin 1, LC3, and p62. Furthermore, relative to the METH + CBD group, the METH + CBD + Eptapirone group exhibited a further significant reduction in the relative fluorescence intensity of Beclin 1, LC3, and p62 (Figure 7A–D). These results indicate that when the classic 5-HT1A agonist Eptapirone is co-administered, CBD (a positive allosteric modulator of 5-HT1A) and Eptapirone may exert a synergistic effect, thereby enhancing the intervention efficacy of CBD. When WAY-100635 was applied, it was observed that compared with the METH + CBD group, the METH + CBD + WAY-100635 group showed significantly increased expression levels of Beclin 1, LC3, and p62 (Figure 7E–H). This suggests that administration of WAY-100635 abolishes the intervention effect of CBD, leading to exacerbated autophagy.

These findings further confirm that CBD inhibits METH-induced neuronal autophagy through the 5-HT1A signaling pathway, with 5-HT1A serving as a key mediator in this process.

## 3. Discussion

METH, a widely abused psychostimulant globally, has been the subject of extensive research regarding its neurotoxicity [30]. In recent years, accumulating evidence has indicated that METH exposure induces autophagic responses in cells, both in vivo and in vitro [31,32]. In previous related studies, activation of microglia, a significant increase in microglial number, and elevated expression of LC3-II and Beclin-1 were observed in the human striatum of METH-abusing individuals and those with HIV + METH comorbidity. Additionally, METH significantly upregulated the expression of autophagy-related proteins (LC3-II, Beclin-1, ATG5, and ATG7) in microglia and the striatum of C57BL/6J mice [33]. Other studies revealed that METH exposure substantially increased autophagy levels in the rat striatum and SH-SY5Y cells [34]. Using primary neuronal cells from tree shrews as subjects, related research demonstrated that METH alone or in combination with HIV-Tat treatment significantly increased the protein expression of autophagy-related genes (Beclin-1, LC3B, ATG5, and ATG7), accompanied by enhanced autophagic flux. Furthermore, transmission electron microscopy confirmed the presence of autophagosomes in response to METH or HIV-Tat treatment [35]. In the present study, we used primary neurons from SD rats to investigate the effect of METH on autophagy levels. Our findings showed that METH reduced neuronal viability and significantly elevated autophagy levels in neurons. The autophagy level exhibited dose- and time-dependent relationships with METH, as validated by increased expression of Beclin 1, LC3, and p62, as well as enhanced autophagic flux. These results are consistent with previous studies indicating that METH can upregulate cellular autophagy levels [33]. Of note, the simultaneous elevation of LC3-II and p62 indicates defective lysosomal degradation during autophagy. Given the absence of lysosomal inhibitor treatments in our experimental design, we cautiously interpret the data as indicating disordered autophagic activity in METH-exposed neurons.

Studies have shown that activation of 5-HT receptors inhibits autophagy, leading to decreased Beclin-1 expression and a reduced LC3-II/LC3-I ratio [13]. In mice, intracerebral injection of selective 5-HT receptor agonists reduces hippocampal Beclin-1 expression and alters the LC3-II/LC3-I ratio, thereby regulating hippocampal autophagy and promoting the extinction of contextual fear memory [36]. In animal models, prolonged exposure to stressors simultaneously upregulates 5-HT2 receptors [37] and downregulates 5-HT1A receptors [38]. These findings indicate that 5-HT is involved in the regulation of autophagy. Our results demonstrated that METH exposure significantly reduced 5-HT1A expression in neurons. To clarify the role of 5-HT1A in METH-induced autophagy, we therefore investigated the effects of 5-HT1A activation and antagonism. Eptapirone (F11440), a potent and selective 5-HT1A receptor agonist with proven roles in anti-anxiety and antidepressant effects, was used in this context [27]. Consistent with previous studies showing that activation of 5-HT agonists significantly reduces autophagy-related protein expression [13], our analysis of autophagy-related proteins after Eptapirone intervention revealed that this agonist significantly attenuated METH-induced autophagy. Conversely, treatment with the 5-HT1A antagonist WAY-100635 [39] exacerbated autophagy in neurons. These results suggest that 5-HT1A is involved in METH-induced neuronal autophagy.

5-HT is an inhibitory neurotransmitter, and its 5-HT1A receptor is negatively coupled to the downstream AC/cAMP/PKA/CREB signaling pathway [40,41]. It has been reported that administration of METH during adolescence significantly reduces the expression level of 5-HT1A in male mice [42]. Additionally, METH intake leads to a significant increase in the mRNA expression level of AC in the cerebral cortex [43,44]. As a key kinase for CREB phosphorylation, PKA enables CREB to play an important role in drug addiction. Studies have shown that METH increases cAMP concentration and CREB phosphorylation in lipopolysaccharide-activated microglia, and this effect of METH is blocked by the PKA antagonist H89 [45]. In rats with long-term morphine administration, the PKA/CREB signaling pathway is activated, and the protein expression levels of p-PKA and p-CREB are significantly increased [46]. These studies suggest that the 5-HT1A/AC/cAMP/PKA/CREB signaling pathway is involved in neurotoxicity induced by addictive drugs. We therefore hypothesized that the 5-HT1A/AC/cAMP/PKA/CREB signaling pathway regulates METH-induced autophagy and conducted further investigations. The results showed that METH exposure significantly reduced 5-HT1A expression while markedly increasing the expression of all molecules in the AC/cAMP/PKA/CREB signaling pathway. Administration of Eptapirone significantly reversed these METH-induced changes, leading to a notable decrease in the expression of molecules in the AC/cAMP/PKA/CREB pathway. These results suggest that the 5-HT1A/AC/cAMP/PKA/CREB signaling pathway may be involved in METH-triggered autophagy, further supporting the validity of our hypothesis.

The chemical components extracted from cannabis are primarily cannabinoids, including delta-9-tetrahydrocannabinol (THC) and CBD. Among the various components of cannabis, THC exhibits strong addictive properties and is a key factor contributing to cannabis abuse. In contrast to THC, which has psychoactive effects, CBD is non-addictive [47] and possesses anti-inflammatory, antioxidant, and neuroprotective properties. CBD has been demonstrated to have therapeutic effects on neuropsychiatric disorders, including substance use disorders [48,49,50], with particular promise in the treatment of drug addiction [51]. It has also been reported that CBD exerts therapeutic effects in METH addiction [52,53]. Previous studies have demonstrated that CBD can modulate METH-induced conditioned place preference in rats [54], reduce METH’s rewarding effects [55], and decrease rats’ motivation to self-administer METH [56]. Additionally, CBD has been shown to exert a protective effect against METH-induced cardiotoxicity, with evidence suggesting a link between CBD and the PKA/CREB signaling pathway. Collectively, these findings indicate a close association between CBD and METH [57]. Based on this, we hypothesized that CBD is involved in METH-induced autophagy and that the 5-HT1A/AC/cAMP/PKA/CREB signaling pathway mediates this process. Our results showed that CBD treatment significantly reduced the expression levels of Beclin 1, LC3, and p62, indicating that CBD effectively alleviates METH-induced autophagy—with 1 μM and 10 μM CBD exerting the most pronounced effects. Similarly, analysis of molecules related to the 5-HT1A/AC/cAMP/PKA/CREB signaling pathway following CBD treatment revealed that, like Eptapirone, CBD reverses METH-induced alterations in this pathway. Interestingly, 0.1 μM CBD only partially downregulates proteins in the 5-HT1A/AC/cAMP/PKA/CREB pathway, which may be closely associated with a dose-dependent mechanism [58]. From the perspective of molecular action, low-concentration CBD may only partially activate the 5-HT1A receptor or interact weakly with it, resulting in insufficient inhibition of the downstream AC/cAMP/PKA/CREB pathway. For instance, in the experiment, 0.1 μM CBD only downregulates cAMP, PKA, and p-CREB but exerts no significant effect on other molecules such as AC and CREB, suggesting that its regulation of the pathway may not reach the threshold required for comprehensive inhibition. Regarding changes in autophagy-related proteins, low-concentration CBD may fail to effectively reverse METH-induced upregulation of LC3 and p62. In contrast, 1 μM and 10 μM CBD can comprehensively downregulate pathway molecules and inhibit autophagy, indicating that CBD must reach a certain concentration to fully exert its neuroprotective effect. This aligns with the dose-dependent law of drug action—higher concentrations are more likely to saturate target binding sites, thereby producing a more significant regulatory effect [59]. Additionally, the partial effect of 0.1 μM CBD may be related to the compensatory mechanism of intracellular signal transduction. At low doses, the pathway is not completely blocked, and residual active molecules can still maintain a partially activated state of autophagy [60]. This phenomenon supports the specificity of CBD’s action through the 5-HT1A pathway and provides an experimental basis for subsequent exploration of its dose–effect relationship.

**Figure 8:**
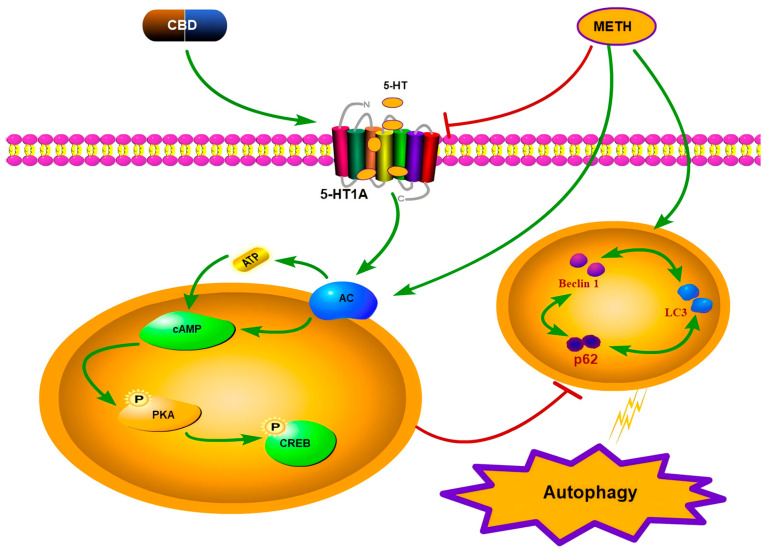
Mechanism of CBD alleviating METH-induced neuronal autophagy via the 5-HT1A/AC/cAMP/PKA/CREB pathway. Green arrows indicate activation/promoting regulatory effects; red blunt-ended lines denote inhibition/antagonistic effects.

Notably, CBD intervention alone did not increase 5-HT1A expression. Previous studies have shown that 5-HT1A, a 5-HT receptor coupled to Gi/o proteins, is associated with the anti-aversive and other pharmacological effects of cannabinoids, including their neuroprotective effects. A pioneering in vitro study by Russo and colleagues indicated that CBD can act as a 5-HT1A receptor agonist, promoting 5-HT1A-mediated neurotransmission [61]. Subsequent to this preliminary study, research has confirmed that the anxiolytic effect of CBD depends on the promotion of 5-HT1A-mediated neurotransmission [62]. However, another study noted that this agonist effect has not been validated in subsequent investigations [63]. Thus, CBD may not be a 5-HT1A receptor agonist as initially proposed. Although the specific mechanism remains unclear, this suggests that CBD’s effect on 5-HT1A activation may result from allosteric interactions with the receptor’s binding site and/or interference with intracellular pathways [64,65]. In this study, CBD treatment alone did not increase the basal expression level of 5-HT1A but significantly reversed METH-induced downregulation of 5-HT1A. This suggests that CBD does not act through a classic agonist mode (e.g., directly activating the receptor and upregulating its expression). Given the allosteric regulatory properties of GPCRs [66], we hypothesize that CBD may bind to the allosteric site of the 5-HT1A receptor, enhance the affinity of its endogenous ligands (such as serotonin), or stabilize the receptor’s active conformation, thereby restoring its inhibitory function on the downstream AC/cAMP/PKA/CREB pathway. Multiple studies have demonstrated that CBD exerts anti-invasive and anti-nociceptive effects, with the ability to protect the 5-HT1A receptor [67,68]. Meanwhile, the binding of CBD to the 5-HT1A receptor mediates its protective, anti-invasive, and anti-nociceptive actions [67,68]. Intriguingly, our study found that the combination of CBD and eptapirone was more effective in reducing autophagy than eptapirone alone, suggesting a potential synergistic effect between the two compounds that enhances the intervention efficacy of CBD [69,70]. Additionally, in research related to drug addiction, pretreatment with WAY-100135 blocked the effects of CBD on addictive substances, confirming the critical role of 5-HT1A in the mechanism of action of CBD [69,71]. In the present study, co-administration of WAY-100635 reversed the therapeutic effects of CBD, further indicating an association between CBD and 5-HT1A. These findings suggest that CBD may mitigate METH-induced cellular autophagy by modulating the 5-HT1A/AC/cAMP/PKA/CREB signaling pathway (Figure 8).

This study was conducted entirely using in vitro primary rat hippocampal neurons, and no in vivo animal experiments were performed. As a result, the translational value of our findings for clinical application is limited to a certain extent. In vitro cell models can effectively clarify intracellular molecular mechanisms with high controllability, but they cannot simulate the complex microenvironment, systemic metabolism and inter-tissue interactions of living animals. Despite this limitation, in vitro cellular experiments are an essential foundation for exploring the preliminary molecular mechanisms of drugs. In our follow-up research, we will carry out systematic in vivo animal experiments to further verify the regulatory effect of CBD on the 5-HT1A/AC/cAMP/PKA/CREB pathway and METH-induced neuroautophagy and evaluate the in vivo efficacy and safety of CBD, so as to improve the translational potential of this research. In addition, this study mainly focuses on the 5-HT1A-mediated signaling pathway. We cannot rule out the contributions of other well-characterized CBD targets, including CB1, CB2 and TRPV1 receptors, which are also known to participate in neuronal protection and stress responses. Further research is needed to clarify the potential crosstalk between 5-HT1A and these receptors in the context of METH neurotoxicity.

In conclusion, CBD may be a promising candidate for inhibiting the abuse and rewarding effects of addictive drugs as well as for treating neurotoxicity, offering a new perspective for future therapeutic strategies.

## 4. Materials and Methods

### 4.1. Animals

A total of 20 Sprague-Dawley rats (6 weeks old, weight: 180~220 g, 10 males and 10 females) were purchased from the Laboratory Animal Center of Kunming Medical University. These rats were used to breed suckling rats for primary neuron extraction. All animal experiments were conducted in accordance with the “KMU Guidelines for the Care and Use of Experimental Animals” and were approved by the KMU Animal Care and Use Ethics Committee (approval code: kmmu2020403). Rats were housed in a standard environment (room temperature 22 ± 1 °C, humidity 50~60%), with males and females caged at a 1:1 ratio, and had free access to food and water.

### 4.2. Drugs

METH (purity > 98%) was lawfully obtained from the Yunnan Public Security Bureau (Kunming, Yunnan, China). It was solubilized in physiological saline to a concentration of 100 mM/mL [33]. CBD (purity: 99.0%, Push Bio-Technology, PD020435, Chengdu, China) was dissolved in a saline vehicle containing 5% dimethyl sulfoxide (DMSO) and 5% polysorbate 80 (Tween-80) to a concentration of 10 mM/mL [72]. Eptapirone (HY-19946) and WAY-100635 (HY-10349) were purchased from MedChemExpress (Monmouth Junction, NJ, USA). The chemical structures of these compounds are presented in Supplementary Figure S2.

### 4.3. Primary Neuron Extraction and Culture

The brains of 24 h old neonatal SD rats were dissected and transferred to Petri dishes containing DMEM medium supplemented with 1% penicillin/streptomycin (Solarbio Life Sciences, P1400, Beijing, China). Hippocampal neurons were collected and washed twice with precooled phosphate-buffered saline (PBS) (Solarbio Life Sciences, P1020, Beijing, China). An appropriate amount of 0.25% trypsin (Gibco, 25200072, Waltham, MA, USA) was added for 10 min of digestion, followed by the addition of DMEM medium (Gibco, 11995073, Waltham, MA, USA) containing 5% fetal bovine serum (Gibco, A5670801, Waltham, MA, USA) to terminate digestion. The isolated cells were uniformly seeded in Neurobasal medium (STEMCELL, 05790, Vancouver, BC, Canada) supplemented with B27 (1:50, STEMCELL, 05711, Vancouver, BC, Canada), 200 mM L-glutamine (Gibco, 25030081, Waltham, MA, USA) and 1% penicillin–streptomycin. The seeded cells were cultured in a 37 °C, 5% CO_2_ cell incubator, with the medium changed daily [35]. After 7~8 days of culture, neurons were identified by immunofluorescent labeling using the neuronal phenotypic marker MAP2 [73] (Supplementary Table S2) and they were confirmed to meet the experimental requirements (Supplementary Figure S1). Each 75 cm^2^ culture flask contained hippocampal neurons isolated from the brain tissues of 3 neonatal rats, with three flasks per group (n = 3) to ensure sufficient biological replicates for subsequent drug treatments and molecular detection.

### 4.4. Drug Treatments

To screen the optimal concentration and time point for METH-induced neuronal autophagy, neurons were treated with METH at concentrations of 0.5, 1, 2, and 4 mM for 24 h, or with 2 mM METH for 3, 6, 12, 24, and 48 h, respectively. These concentrations and time points were referenced from previous studies [74]. For CBD treatment, neurons were first exposed to METH for 24 h, and then post-treated with 0.1, 1, and 10 μM CBD for 1 h; the CBD concentrations were based on our team’s previous research [75]. As per previous studies, cells were pretreated with 10 μM Eptapirone [76] or 0.5 μM WAY-100635 [77] for 1 h, followed by METH treatment for 24 h, with CBD administered as a post-treatment after METH exposure.

### 4.5. Western Blot

Cells were collected and centrifuged at 12,000 rpm at 4 °C for 15 min. The pellet was retained, the samples were shattered by ultrasound in a lysis buffer (Epizyme Biotech, PC101, Shanghai, China) and lysed for 30 min at 4 °C. Protein concentration was determined using a BCA assay kit (Epizyme Biotech, ZJ102, Shanghai, China). After adding protein loading buffer, the solution was heated at 99 °C for 10 min. The denatured proteins (25 μg) were separated by 12% SDS-PAGE (Epizyme Biotech, PG213, Shanghai, China) and transferred onto a 0.45 μm polyvinylidene difluoride (PVDF) membrane (Sigma-Aldrich, IPVH00010, St. Louis, MO, USA). The membrane was blocked with 5% skim milk in TBST (0.1% Tween 20 in Tris-buffered saline) at room temperature for 2 h, then incubated with primary antibodies (Supplementary Table S1) (diluted in 5% skim milk) at 4 °C overnight. The next day, the membrane was washed 3 times with TBST (15 min each) and then incubated with secondary antibodies (Supplementary Table S1) (diluted in 5% skim milk) at room temperature for 1.5 h. Signals were detected using an enhanced chemiluminescence (ECL) kit (Biosharp Life Sciences, BL520B, Beijing, China), and images were captured with a ChemiDoc MP system (Bio-Rad Laboratories, ChemiDoc MP, Hercules, CA, USA). Protein levels were analyzed using ImageJ v1.46r software and normalized to β-actin as the internal reference. For the detection of the internal reference β-actin, we used standard membrane stripping and reprobing procedures after target protein detection. No cross-contamination was observed during the whole process.

### 4.6. Immunofluorescence Staining

Cell climbing slices were fixed with 4% paraformaldehyde for 30 min and washed three times with PBS. PBS containing 0.2% Triton X-100 (Solarbio Life Sciences, T8200, Beijing, China) was added; after incubation for 30 min, the slices were washed three times with PBS. A 10% goat serum (Solarbio Life Sciences, SL038, Beijing, China) blocking solution was added for blocking at room temperature for 1 h. The blocking solution was aspirated, and primary antibody dilutions (Supplementary Table S2) prepared with PBS were added, followed by incubation at 4 °C overnight. The next day, the primary antibodies were discarded, and the slices were washed three times with PBS. Then, under light protection, secondary antibody dilutions (Supplementary Table S2) prepared with PBS were added and incubated at 37 °C for 1 h in the dark, followed by three washes with PBS. Finally, the slices were mounted using DAPI-containing mounting medium (Solarbio Life Sciences, S2110, Beijing, China). Images were captured using a fluorescence microscope (Leica, Model DMi8, Wetzlar, Germany), and fluorescence intensity was analyzed using ImageJ v1.46r software.

### 4.7. Cell Viability Assay

Cell viability was assessed using a CCK-8 kit (a colorimetric assay for detecting cell viability and proliferation) (Absin, abs50003, Shanghai, China). Briefly, neurons were seeded into 96-well plates (NEST Biotechnology, 713011, Wuxi, China) and co-incubated with the drugs. After treatment, 10% CCK-8 reagent was added to each well, followed by incubation at 37 °C for 60 min. The absorbance was then measured at 450 nm using a microplate reader.

### 4.8. Transfection with Ad-mCherry-GFP-LC3B

Neurons were seeded on glass bottom dishes and cultured until 80% confluent. Neurons were transfected with Ad-mCherry-GFP-LC3B (adenovirus vector carrying mCherry red fluorescent tag) [35,78] (adenovirus expressing mCherry-GFP-LC3B fusion protein) (Beyotime, C3011, Shanghai, China) at 20 MOI for 24 h, followed by treatment with METH and CBD. After an additional 24 h of culture, cells were fixed with 4% paraformaldehyde and visualized using a fluorescence microscope (Leica, Model DMi8, Wetzlar, Germany), as previously described.

### 4.9. Data Analysis

Statistical analyses of experimental data were performed using SPSS v26.0 software. All experimental data in the figures and tables are presented as means ± standard deviation (SD). For comparisons between two groups, an independent samples *t*-test was used; one-way analysis of variance (ANOVA) was applied to the screening experiments for METH concentration and action time; two-way ANOVA (without repeated measures) was used for analyses among other multiple groups. Tukey’s HSD post hoc tests were conducted after all ANOVA analyses. All data were confirmed to conform to a normal distribution and homogeneity of variance. The level of significance was set to *p* < 0.05.

## 5. Conclusions

This study confirms that the regulation of autophagic activity in primary neurons by CBD may be associated with its intervention on the 5-HT1A receptor and its downstream signaling pathway. Specifically, CBD can alleviate abnormal autophagy by restoring 5-HT1A expression and inhibiting the excessive activation of its downstream pathways, thereby exerting a neuroprotective effect. This research may provide innovative insights for formulating effective anti-drug intervention strategies.

## Data Availability

The original contributions presented in this study are included in the article and Supplementary Materials. Further inquiries can be directed to the corresponding authors.

## Supplementary Materials

The following supporting information can be downloaded at https://www.mdpi.com/article/10.3390/ijms27135677/s1.

## Abbreviations

AC, adenylate cyclase; ATG5, autophagy-related gene 5; ATG7, autophagy-related gene 7; CBD, Cannabidiol; CNS, central nervous system; CREB, cAMP response element-binding protein; cAMP, cyclic adenosine monophosphate; ERK1/2, extracellular regulated protein kinases 1/2; GPCRs, G protein-coupled receptors; HIV, human immunodeficiency virus; LC3, microtubule-associated protein light chain 3; MAP2, microtubule-associated protein 2; METH, Methamphetamine; p-CREB, phosphorylated cAMP response element-binding protein; p-PKA, phosphorylated protein kinase A; PKA, protein kinase A; SD, Sprague-Dawley; THC, delta-9-tetrahydrocannabinol; 5-HT, 5-hydroxytryptamine; 5-HT1A, 5-hydroxytryptamine 1A receptor.
