# Supplementary material for: Cannabidiol Attenuates Methamphetamine-Induced Autophagy in Primary Rat Neurons via the 5-HT1A/AC/cAMP/PKA/CREB Signaling Pathway
Source: Int J Mol Sci. 2026 Jun 24;27(13):5677. doi: 10.3390/ijms27135677 (PMC13362501; doi:10.3390/ijms27135677)
Supplement: Supplementary file 1 [file ijms-27-05677-s001.zip › ijms-4345199-supplementary.pdf]

Cannabidiol attenuates methamphetamine-induced autophagy in primary rat neurons via the 5-HT1A/AC/cAMP/PKA/CREB signaling pathway

Xiong Li [†], Jiameng Ding [†], Xiao Ma, Dongxian Zhang [*]

Supplementary Table S1. Antibody catalogue-Western blotting

Antibody	Host	Distributor	Working dilution
**Primary antibody**			
Beclin 1	R	Proteintech (11306-1-AP)	1:1000
LC3	R	Abcam (ab192890)	1:1000
p62	R	Abcam（ab211324）	1:1000
5-HT1A	R	Abcam (ab85615)	1:1000
AC	R	Abcam (ab125093)	1:1000
cAMP	R	Abcam (ab76238)	1:1000
PKA	R	Proteintech (24503-1-AP)	1:1000
p-PKA	R	Abcam (ab75991)	1:1000
CREB	R	Proteintech (12208-1-AP)	1:1000
p-CREB	R	CST (9198s)	1:1000
β-actin	M	Proteintech (66009-1-Ig)	1:10000
**Secondary antibody**			
Anti-Mouse HRP G	M	Proteintech (SA00001-1)	1:10000
Anti-Rabbit HRP G	R	Proteintech (SA00001-2)	1:5000

Supplementary Table S2. Antibody catalogue-Immunofluorescence staining

Antibody	Host	Distributor	Working dilution
**Primary antibody**			
Beclin 1	R	Proteintech (11306-1-AP)	1:200
LC3	R	Abcam (ab192890)	1:200
p62	R	Abcam（ab211324）	1:200
**Secondary antibody**			
Goat Anti-Mouse. 594	M	Abbkine (A23410)	1:200
Goat Anti-Rabbit. 488	R	Abcam (ab150077)	1:200

Supplementary Figure S1

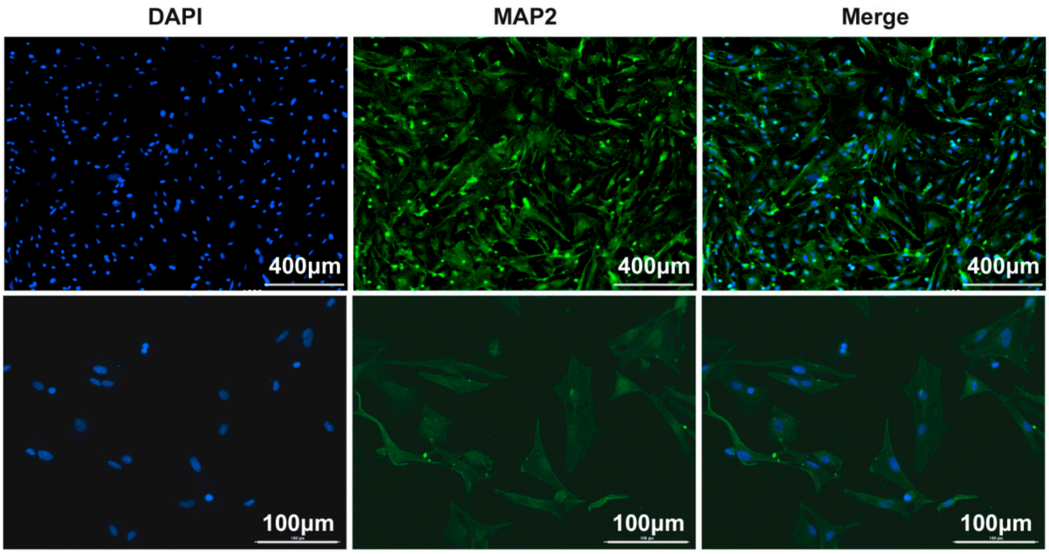

Figure S1. Identification of primary neurons: Representative immunofluorescence images of the neuronal marker MAP2. Scale bar = 400 or100 μm.

Supplementary Figure S2

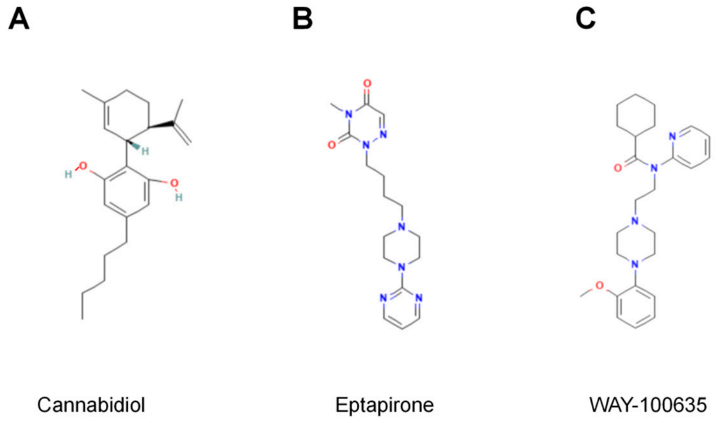

Figure S2. Chemical structural formula. (**A**) Cannabidiol. (**B**) Eptapirone. (**C**) WAY-100635.
